# The Influence of Temperature Increase on the Toxicity of Mercury Remediated Seawater Using the Nanomaterial Graphene Oxide on the Mussel *Mytilus galloprovincialis*

**DOI:** 10.3390/nano11081978

**Published:** 2021-07-31

**Authors:** Francesca Coppola, Amadeu M. V. M. Soares, Etelvina Figueira, Eduarda Pereira, Paula A. A. P. Marques, Gianluca Polese, Rosa Freitas

**Affiliations:** 1 Department of Biology CESAM, University of Aveiro, 3810-193 Aveiro, Portugal; c.francesca@ua.pt (F.C.); asoares@ua.pt (A.M.V.M.S.); efigueira@ua.pt (E.F.); 2 Department of Chemistry LAQV-REQUIMTE, University of Aveiro, 3810-193 Aveiro, Portugal; eduper@ua.pt; 3 Department of Mechanical Engineering TEMA, Universidade de Aveiro, 3810-193 Aveiro, Portugal; paulam@ua.pt; 4 Department of Biology, University of Naples Federico II, 80126 Naples, Italy; gianluca.polese@unina.it

**Keywords:** metals, warming, bivalves, oxidative stress, nanomaterial, histopathology

## Abstract

Mercury (Hg) has been increasing in waters, sediments, soils and air, as a result of natural events and anthropogenic activities. In aquatic environments, especially marine systems (estuaries and lagoons), Hg is easily bioavailable and accumulated by aquatic wildlife, namely bivalves, due to their lifestyle characteristics (sedentary and filter-feeding behavior). In recent years, different approaches have been developed with the objective of removing metal(loid)s from the water, including the employment of nanomaterials. However, coastal systems and marine organisms are not exclusively challenged by pollutants but also by climate changes such as progressive temperature increment. Therefore, the present study aimed to (i) evaluate the toxicity of remediated seawater, previously contaminated by Hg (50 mg/L) and decontaminated by the use of graphene-based nanomaterials (graphene oxide (GO) functionalized with polyethyleneimine, 10 mg/L), towards the mussel *Mytilus galloprovincialis*; (ii) assess the influence of temperature on the toxicity of decontaminated seawater. For this, alterations observed in mussels’ metabolic capacity, oxidative and neurotoxic status, as well as histopathological injuries in gills and digestive tubules were measured. This study demonstrated that mussels exposed to Hg contaminated seawater presented higher impacts than organisms under remediated seawater. When comparing the impacts at 21 °C (present study) and 17 °C (previously published data), organisms exposed to remediated seawater at a higher temperature presented higher injuries than organisms at 17 °C. These results indicate that predicted warming conditions may negatively affect effective remediation processes, with the increasing of temperature being responsible for changes in organisms’ sensitivity to pollutants or increasing pollutants toxicity.

## 1. Introduction

### 1.1. Impacts of Mercury in Marine Ecosystems

Among hazardous chemical elements, mercury (Hg) is on the top ten list of toxic contaminants in the world [1,2]. Mercury is ubiquitous in waters, sediments, soils and air, and can be originated from natural events, such as erosion and volcanic eruptions [3,4,5]. However, the presence of this element in the environment, especially in the aquatic systems, results mostly from anthropogenic activities such as the burning of fossil fuels, gold mining and, more recently, improper disposal of electronic products [6,7,8,9,10]. In aquatic environments, especially marine coastal systems (estuaries and lagoons), most of the metal(loid)s, including Hg, have the capacity to be associated with sediments and to be present in the water column and biota tissues [4,11,12,13]. Although in open seawater Hg concentration ranges from 0.5 to 3.0 ng/L [14], Hg was detected in concentrations up to 27 µg/L in coastal waters [15]. Moreover, Sunderland et al. [13] highlighted that at the current emission rate, Hg concentrations in the North Pacific Ocean would rise by 50% in 2050 compared to levels recorded in 1995. This situation may happen in other areas around the world since Hg is still commonly used, for example, in new technological applications [7]. The bioavailability of Hg in aquatic environments enhances concerns, namely regarding marine organisms as bivalves due to their lifestyle characteristics, including their sedentary and filter-feeding behavior, which facilitates contaminants accumulation and, consequently, may generate toxicity [16,17,18,19]. Thus, due to their characteristics, bivalves are among the best bioindicator species of environmental pollution [20,21,22,23]. Previous studies showed that, besides their capacity to accumulate Hg (ranging from 0.015 to more than 40 µg/g), exposure to Hg can induce oxidative stress and neurotoxicity, cellular damage and histopathological alterations in bivalves [1,24,25,26,27,28]. 

### 1.2. Strategies to Remediate Contaminated Waters

In the last decade, different methodologies, such as ultrafiltration, reverse osmosis and electrochemical methods, have been developed to remediate polluted waters before being discharged into coastal systems [1,29,30,31,32,33,34,35]. Nevertheless, these methodologies are frequently low-cost but inefficient, or efficient but expensive [36,37,38,39]. To overcome these limitations, alternative methods and materials, especially based on nanostructured materials, have been synthetized and tested [40,41,42,43]. Among several materials, graphitic carbon atoms have demonstrated excellent electrical conductivity, high mechanical strength and thermal conductivity, high impermeability to gases and optical transparency [44,45]. Due to its characteristics, graphene has been used in a vast diversity of applications [46,47,48]. As an example, Bessa et al. [49] synthesized and characterized a new nanomaterial based on graphene oxide (GO) and functionalized it with polyethyleneimine (GO–PEI), which proved to be effective (easy to prepare and low-cost) for removing Hg from seawater in 24 h. This excellent performance was attributed to the synergistic effect resultant from the interactions between GO and PEI, giving a high content of N-rich groups and negative zeta potential over a wide pH range (from 2 to 12). Based on this study, Coppola et al. [1] demonstrated that under control temperature (17 °C), seawater contaminated with Hg (50 µg/L) and remediated using GO–PEI did not present toxic effects in mussels *Mytilus galloprovincialis* after chronic exposure. These authors concluded that GO‒PEI was able to significantly reduce the concentration of Hg in seawater, being safe to wildlife if discharged into aquatic systems. However, Sanchez et al. [50] reported that GO is able to interact with biomolecules causing the generation of ROS (reactive oxygen species) in target cells as a potential mechanism for toxicity. In fact, despite a high hydrophobic surface area, GO may lead to significant interactions with membrane lipids causing direct physical toxicity or adsorption of biological molecules. Regarding the toxic effects of PEI towards aquatic invertebrates, Petersen et al. [51] revealed that PEI coatings increased nanotubes toxicity in *Daphnia magna*.

### 1.3. Impacts of Temperature in Marine Organisms

Coastal ecosystems and marine biological resources are not exclusively at risk due to pollution but also to natural pressures, including daily and seasonal temperature changes, with predicted scenarios indicating an increase of seawater temperature up to 2 °C until the end of the century [52,53]. Associated with the temperature rise in aquatic systems, different authors have shown deleterious effects in the inhabiting wildlife, including bivalves. In particular, studies revealed that exposure to warming conditions leads to perturbations on bivalves’ physiological performance, including reduced aerobic scope and the energy available for fitness-related functions, impacts on shell growth and mortality [54,55,56,57,58]. Furthermore, changes in bivalves’ metabolic capacity, oxidative status and neurotoxicity were revealed in bivalves exposed to temperature rise [59,60,61,62,63,64,65]. In addition, the interaction between the increase of temperature and contaminants can affect the bivalve’s sensitivity, changing their vulnerability towards each stressor, but may also change pollutants bioavailability and toxicity [26,60,66,67,68,69,70]. For example, Pirone et al. [71] showed that *M. galloprovincialis* presented higher oxidative stress and cellular damage when exposed to the combination of lead (Pb) and warming conditions in comparison to those mussels under each single stressor. 

Considering the lack of information regarding the impacts that remediated seawater may induce to aquatic wildlife, the present study aimed to: (i) evaluate the toxic effects induced in the mussel *M. galloprovincialis* by remediated seawater (previously contaminated with Hg and remediated by GO‒PEI); (ii) assess the influence of temperature on the toxicity of remediated seawater.

## 2. Materials and Methods

### 2.1. Experiment Setup

Adult mussels of the species *Mytilus galloprovincialis* were collected in the Ria de Aveiro lagoon (Portugal) during low tide at the end of August 2019. More than one hundred mussels were collected, which were transported to the laboratory using plastic containers filled with seawater from the sampling site. Specimens presented a mean body weight of 13.1 ± 2.1 g, mean length of 5.7 ± 0.68 cm and a mean width of 3.0 ± 0.42 cm. In the laboratory, mussels were maintained for one week at a constant temperature, pH and salinity (17 °C, pH 8.10 and 30, respectively) (depuration). The artificial seawater (salinity 30 ± 1, prepared with Tropic Marin^®^ SEA SALT dissolved in osmose water) was renewed every 2 days during this week. Afterward, organisms were divided into two groups and placed in two different climatic rooms: one exposed at 17 ± 1 °C (identified as control; CTL) and the other at 21 ± 1 °C (representing temperature rise) for acclimation during an extra week. A temperature of 21 °C was selected to resemble predicted warming conditions considering projections by IPCC [53,72]. After acclimation, bivalves were exposed for 28 days at different treatments, including: CTL-clean seawater (at 17 and 21 °C; CTL 17 and CTL 21, respectively); Hg, seawater containing Hg (50 g/L, 21 °C); GO–PEI, graphene oxide (GO) functionalized with polyethyleneimine, (10 mg/L, 21 °C); GO–PEI + Hg, GO–PEI and Hg (at 21 °C); RSW, seawater after remediation (at 21 °C) (Table 1). For each treatment, three aquaria (3L) were used, with five individuals per aquarium. During acclimation and exposure periods, animals were fed (with Algamac protein plus (150.000 cells/animal/day)) every other day and maintained in artificial seawater at pH 8.1, photoperiod 12 h light and 12 h dark, and constant aeration. 

The selected Hg concentration (50 µg/L) used to resemble contaminated water (treatment: Hg) was based on the permitted concentration of this metal in wastewater [73] and previous studies testing the capacity of GO–PEI to remove Hg from contaminated water [49]. Considering that in the aquatic ecosystems, most of the Hg is found in the inorganic form (Hg) [74], in the present study, the inorganic form Hg (NO₃)₂ (Sigma Aldrich, St. Louis, MI, USA) was used. A certified standard solution of Hg was used (1000 ± 2 mg/L of Hg(II) in HNO_3_ 0.5 mol/L, from Merck). The amount of GO–PEI (10 mg/L) used for water remediation was selected according to previous studies where the capacity of this material to remove Hg from seawater was demonstrated [49]. The remediated seawater (treatment: RSW) was prepared as described by Coppola et al. [1]. Throughout an experimental period of 28 days, temperatures, salinity and pH were checked daily as well as mussels’ mortality. During the 28 days of exposure, the seawater was renewed weekly and conditions reestablished, including temperature, salinity and concentrations of the metal and nanomaterial. Furthermore, to compare real Hg exposure concentrations with Hg nominal concentrations, seawater samples from each aquarium were collected immediately after spiking following weekly seawater renewals. After the experimental period (28 days), mussels’ soft tissues were used to analyze and evaluate the Hg concentrations, histopathological alterations, oxidative stress and neurotoxicity. The results obtained were discussed comparing biological responses observed at 17 [1] and 21 °C (present study). For the histological assessment, one mussel from each aquarium (three per treatment) was meticulously opened, and the soft tissue was separated from the shell and fixed in Bouin’s fluid for 24 h at room temperature to analyze gills and digestive glands, as described by Leite et al. [75]. Three organisms from each aquarium (9 per treatment) were frozen in liquid nitrogen for Hg quantifications and biochemical assays. The whole soft tissues from each mussel were homogenized under liquid nitrogen and divided into five aliquots of 0.5 g fresh weight (FW). From each individual, four aliquots were used for biochemical analyses (each one for a specific buffer and respective biomarkers) and one for Hg quantification.

### 2.2. Graphene Oxide with Ethyleneimine Polymer

The GO‒PEI material was synthetized under laboratory conditions, mixing graphene oxide (GO) in water solution (0.4 wt % concentration from Graphenea) with ethyleneimine polymer (PEI) solution 50% (*w*/*v*) in water with M.W. 750000 (Sigma Aldrich) with a ratio of 24% *v*/*v* (GO/polymer) with pH 2, as described by Coppola et al. [1] and Bessa et al. [49]. High Mw (750 k) of highly branched PEI and GO nanosheets (1:3 ratio) were used to produce a hydrogel in aqueous acidic medium. Although the synthesis methodology was quite reproducible, each batch of material was analyzed via Attenuated Total Reflectance Fourier Transform Infrared (ATR–FTIR) in a Bruker Tensor 27 FT–IR spectrometer (Bruker Corporation, Bill Rica, MA, USA). The spectra were recorded between 400 and 4000 cm^−1^, with a resolution of 4 cm^−1^ and 256 scans. The microstructure was evaluated using a scanning electron microscope (FEGSEM HITACHI S4100) to prove the synthesis reproducibility. The capacity of GO–PEI to remove Hg was tested each week during the experiment (for a total of 4 weeks). The water samples from the RSW condition were collected each week after the remediation treatment, and Hg was quantified to validate the remediation process.

### 2.3. Mercury Quantification

The Hg in seawater was analyzed as described by Henriques et al. [44] and Coppola et al. [1] using cold vapor atomic fluorescence spectroscopy (CV–AFS) in a PSA 10.025 Millennium Merlin Hg analyzer, and the results were expressed in µg/L. The concentration of Hg in organisms’ soft tissues was quantified by thermal decomposition atomic absorption spectrometry with gold amalgamation (LECO model AMA–254) following Costley et al. [76] and Coppola et al. [1], and the results were expressed in µg/g.

### 2.4. Biological Responses: Metabolic Capacity, Oxidative Stress and Neurotoxicity Biomarkers

The biochemical markers were performed following the methods described in the Supplementary Materials, including: (i) metabolic capacity—electron transport system activity (ETS) expressed in nmol/min/g FW and determined as reported and modified by De Coen and Janssen [77] and King and Packard [78]; (ii) antioxidant enzymes activity—superoxide dismutase (SOD) and catalase (CAT) expressed in U/g FW and determined following Beauchamp and Fridovich [79] and Johansson and Borg [80], respectively; (iii) biotransformation isoenzymes activity—glutathione-S-transferases (GSTs) expressed in U/g FW and determined as described by Habig et al. [81]; (iv) extent of cellular damage—lipid peroxidation levels (LPO) and protein carbonylation levels (PC) expressed in nmol MDA/g FW and nmol/g FW and determined according to Ohkawa et al. [82] and Mesquita et al. [83], respectively; (v) redox balance—ratio between reduced (GSH) and oxidized (GSSG) glutathione, determined by Rahman et al. [84]; (vi) neurotoxicity—acetylcholinesterase activity (AChE) expressed in nmol/min/g FW and determined following Ellman et al. [85].

### 2.5. Biological Responses: Histopathological Measurements

The fixed tissues used to assess histopathological alterations were processed, as described previously [1,86]. The digestive glands and gills were carefully dissected from mussels. After gradually dehydrated from ethanol 70% to absolute alcohol in graded alcohols and cleared in xylene, each piece was embedded in paraffin and cut with a microtome (7 μm thick for each slide) to evaluate the histological alterations. The evaluation of the histopathological index (*ih*) was done following Leite et al. [75]: six slides (with three sections each) were processed for gills and digestive glands. For each slide, six pictures at 40× magnification were taken (*n* = 36 pictures per mussel’s tissue); for each picture, the presence/absence of the considered histological damage was noted (for gills: hemocytes infiltration, evident enlargement of the central vessel, abundance of lipofuscin aggregates; and for digestive glands: hemocytes infiltration, atrophied, necrosis) giving a score (a) from 0 (none) to 6 (diffuse). The alteration level (w) was given for each damage from 1 (minimum severity) to 3 (maximum severity) based on Costa et al. [87].

### 2.6. Integrated Biomarker Response

The integrated biomarker response (IBR) was calculated to understand the general mussels’ health status, using biomarkers results (ETS, SOD, CAT, GSTs, LPO, PC, GSH/GSSG and AChE), following the Beliaeff and Burgeot [88] method.

### 2.7. Statistical Analysis

All results obtained (Hg concentrations in seawater and mussel’s soft tissues, biochemical markers and histopathological index) were submitted for the statistical analysis using PERMANOVA (permutational multivariate analysis of variance) add-on package for PRIMER v6 software [89]. Pearson correlation was used to perform pairwise comparison, with 9999 permutations. Significance (*p*-value) was calculated using a Monte Carlo test. Significant differences between each pair of treatments were assigned for a *p*-value < 0.05. The null hypothesis (H0) tested the existence of no significant differences among treatments: (i) for Hg concentration in seawater and mussels; (ii) for each biochemical marker; (iii) for histopathological alterations. Significant differences among CTL 17, CTL 21, GO–PEI, GO–PEI + Hg, Hg and RSW treatments are represented in figures with different letters.

### 2.8. Multivariate Analysis

The matrix gathering the histopathological index, biochemical markers as well as Hg concentrations was used to calculate the Euclidean distance similarity matrix, which was simplified through the calculation of the distance among centroids (i.e., the mean position of all the points representing a given treatment). The resulting matrix was submitted to ordination analysis (Principal Coordinates, PCO). In the PCO graph, the variables presenting a correlation higher than 75% were represented by a super imposed vector.

## 3. Results

At the end of the exposure period (28 days), 100% survival was observed.

### 3.1. Mercury Quantification

The nominal Hg concentration and the capacity of GO–PEI to remediate the seawater were checked after each weekly renewal. Hg concentrations measured in water from GO–PEI + Hg and Hg treatments showed similar values to the nominal concentrations (52.1 ± 2.2 and 52.4 ± 2.8 µg/L, respectively). Furthermore, Hg concentration in seawater samples from RSW treatment confirmed the 83% capacity of GO–PEI to remove Hg (Hg concentrations in RSW: 9.6 ± 1.5 µg/L). The concentration of Hg in the seawater samples from CTL 17 and CTL 21 treatments (clean seawater at 17 and 21 °C) as well as in samples from GO–PEI treatment were below the limit of quantification (Table 2).

In the soft tissue of *M. galloprovincialis,* the lowest Hg concentrations were measured in RSW compared with those values obtained in mussels exposed to GO–PEI + Hg and Hg treatments (Table 2). The concentration of Hg in mussels’ tissues showed no significant differences between GO–PEI + Hg and Hg conditions (Table 2). Moreover, mussels exposed to CTL 17, CTL 21 and GO–PEI presented Hg concentrations below 1 µg/g.

### 3.2. Biological Assays: Metabolic Capacity, Oxidative Stress and Neurotoxicity Biomarkers

**Figure 1:**
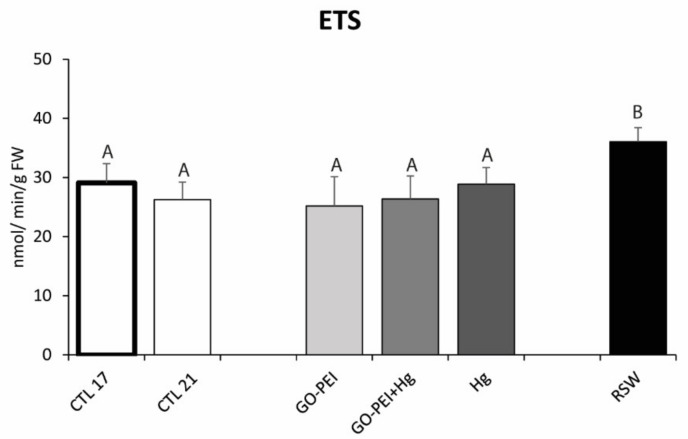
Electron transport system activity (ETS) in *Mytilus galloprovincialis* exposed to different treatments and temperatures (CTL 17, exposure at 17 °C; CTL 21, GO‒PEI, GO–PEI + Hg, Hg and RSW, exposure at 21 °C) at the end of the experiment (28 days). Results are mean ± standard deviation (*n* = 9). Significant differences among the conditions are presented with uppercase letters.

**Figure 2:**
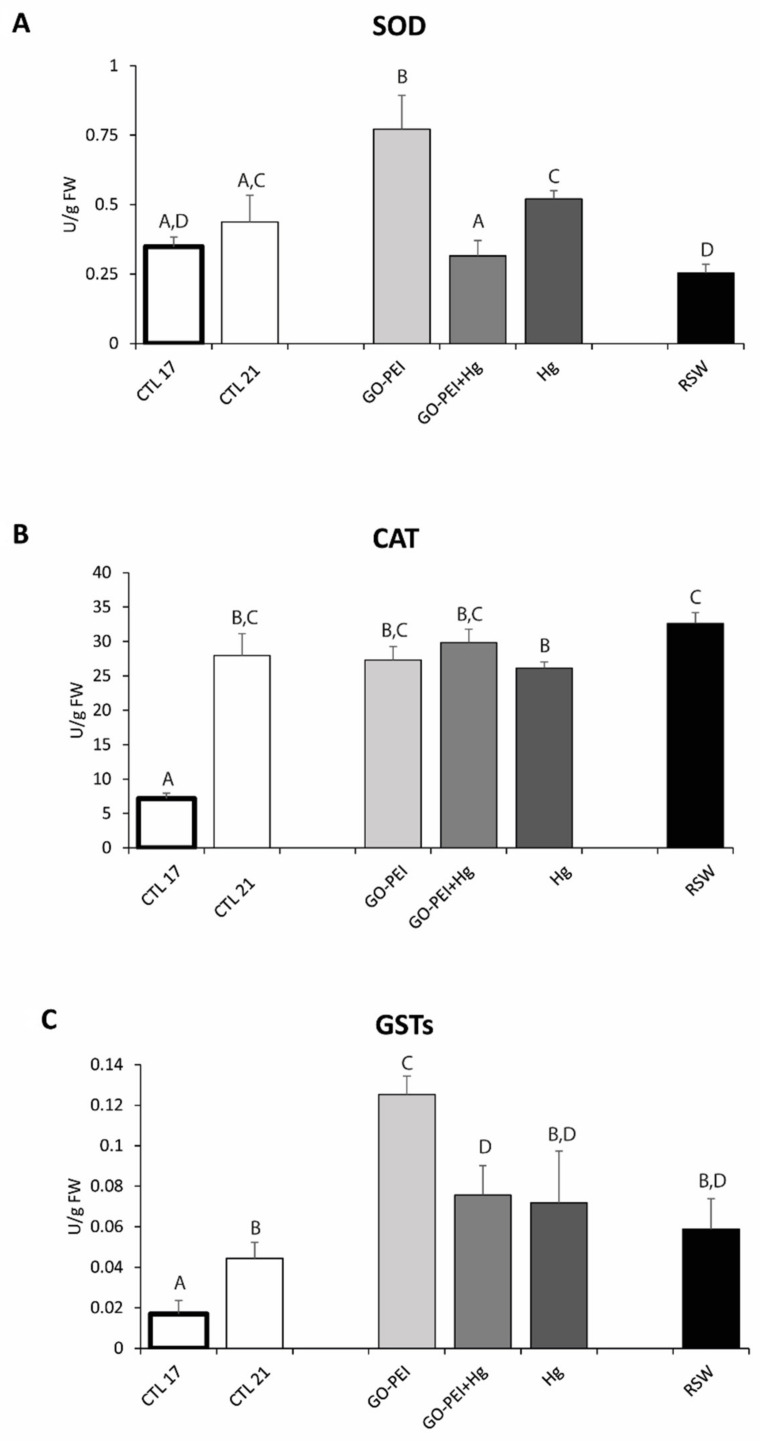
(**A**): Superoxide dismutase activity (SOD); (**B**): catalase activity (CAT); (**C**): glutathione-S-transferases activity (GSTs) in *Mytilus galloprovincialis* exposed to different treatments and temperatures (CTL 17, exposure at 17 °C; CTL 21, GO‒PEI, GO–PEI + Hg, Hg and RSW, exposure at 21 °C) at the end of the experiment (28 days). Results are mean ± standard deviation (*n* = 9). Significant differences among the conditions are presented with uppercase letters.

The ETS activity showed significantly higher values in mussels exposed to RSW compared with mussels exposed to the remaining treatments (Figure 1, Table 3). Significantly higher SOD activity was observed in mussels exposed to GO–PEI compared with the remaining treatments. No significant differences were observed between mussels exposed to CTL 21 and Hg, as well as among mussels exposed to CTL 17, GO–PEI + Hg and RSW (Figure 2A, Table 3). 

The activity of CAT was significantly higher in mussels exposed to 21 °C compared with mussels under 17 °C. Significant differences were also observed between mussels exposed to RSW and exposed to Hg, with higher values in remediated water (Figure 2B, Table 3). Significantly higher GSTs activity was observed in mussels exposed to GO–PEI compared with the remaining treatments. No significant differences were observed among mussels exposed to CTL 21, Hg and RSW as well as among mussels exposed to GO–PEI + Hg, Hg and RSW (Figure 2C, Table 3).

**Figure 3:**
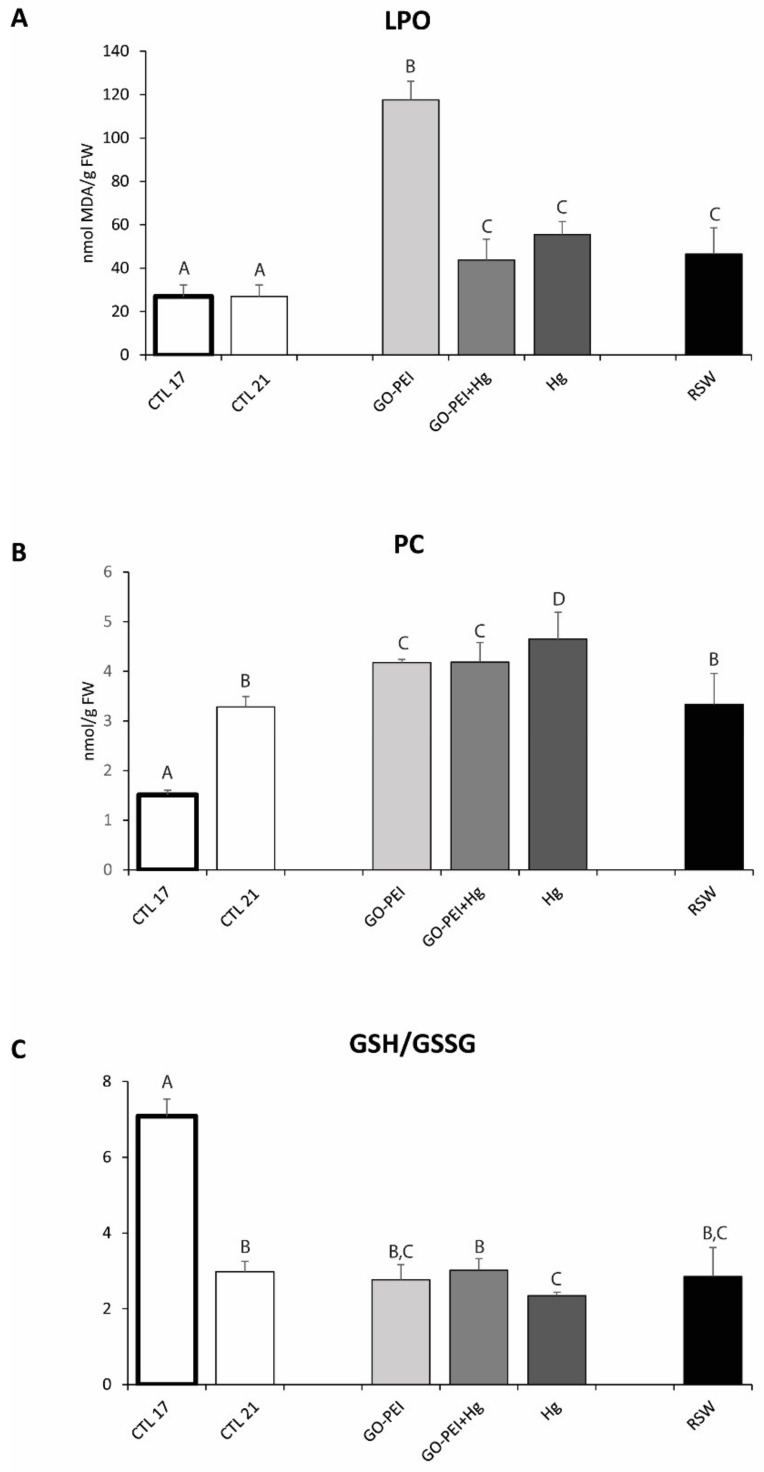
(**A**): Lipid peroxidation levels (LPO); (**B**): protein carbonyl levels (PC); (**C**): ratio between reduced and oxidized glutathione (GSH/GSSG) in *Mytilus galloprovincialis* exposed to different treatments and temperatures (CTL 17, exposure at 17 °C; CTL 21, GO–PEI, GO–PEI + Hg, Hg and RSW, exposure at 21 °C) at the end of the experiment (28 days). Results are mean ± standard deviation (*n* = 9). Significant differences among the conditions are presented with uppercase letters.

Significantly higher LPO levels were observed in mussels exposed to GO–PEI compared with the remaining treatments. No significant differences were observed among mussels exposed to GO–PEI + Hg, Hg and RSW (Figure 3A, Table 3). 

PC levels were significantly higher in mussels exposed to 21 °C compared with mussels under 17 °C. No significant differences were observed between mussels exposed to CTL 21 and RSW, as well as between mussels exposed to GO–PEI and GO–PEI + Hg (Figure 3B, Table 3). GSH/GSSG values were significantly lower in mussels exposed to 21 °C compared with mussels under 17 °C. No significant differences were observed among mussels exposed to CTL 21, GO–PEI, GO–PEI + Hg and RSW (Figure 3C, Table 3).

**Figure 4:**
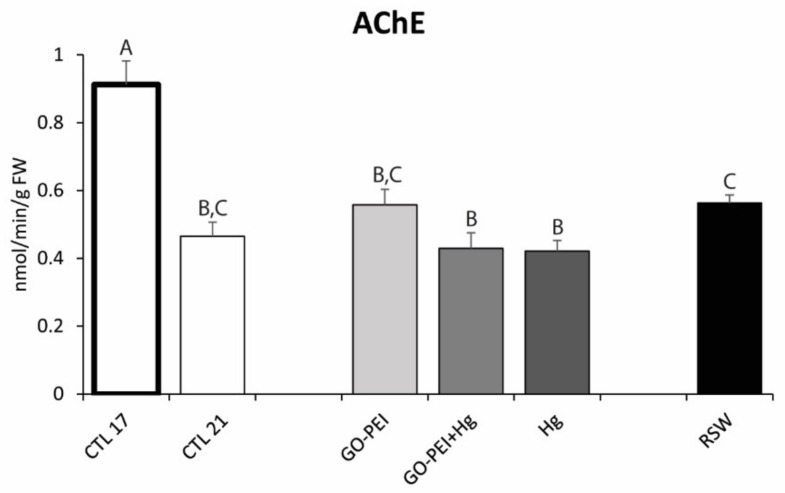
Acetylcholinesterase activity (AChE) in *Mytilus galloprovincialis* exposed to different treatments and temperatures (CTL 17, exposure at 17 °C; CTL 21, GO–PEI, GO–PEI + Hg, Hg and RSW, exposure at 21 °C) at the end of the experiment (28 days). Results are mean ± standard deviation (*n* = 9). Significant differences among the conditions are presented with uppercase letters.

AChE activity was significantly lower in mussels exposed to 21 °C compared with mussels under 17 °C. No significant differences were found among CTL 21, GO–PEI, GO–PEI + Hg and Hg mussels as well as among CTL 21, GO–PEI and RSW mussels (Figure 4, Table 3).

### 3.3. Biological Responses: Histopathological Measurements

**Figure 5:**
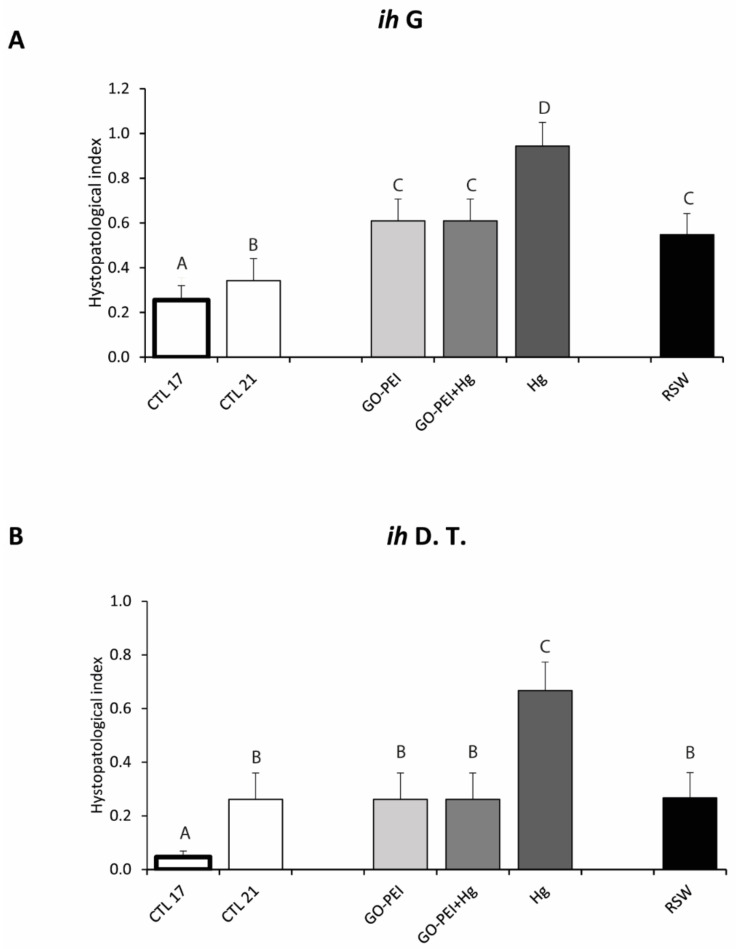
(**A**): Histopathological index in gills (*ih* G); (**B**): histopathological index in digestive tubule (*ih* D.T.) in *Mytilus galloprovincialis* exposed to different treatments and temperatures (CTL 17, exposure at 17 °C; CTL 21, GO–PEI, GO–PEI + Hg, Hg and RSW, exposure at 21 °C) at the end of the experiment (28 days). Results are mean ± standard deviation (*n* = 3). Significant differences between conditions are represented with uppercase letters.

Significantly higher *ih* in gills was observed in organisms exposed to Hg in comparison to mussels exposed to the remaining treatments. No significant differences were found among mussels exposed to GO–PEI, GO–PEI + Hg and RSW (Figure 5A, Table 3).

**Figure 6:**
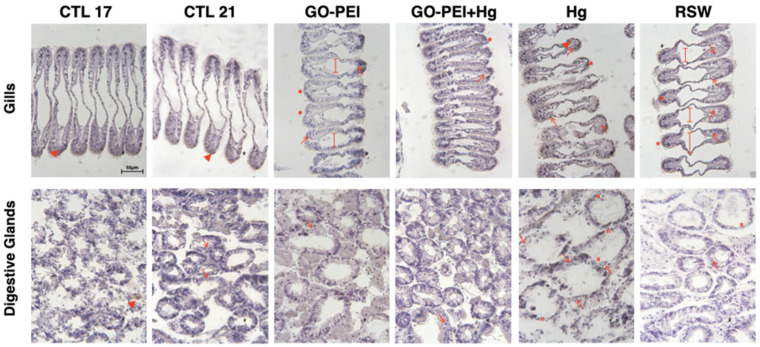
Micrographs of different tissues in *Mytilus galloprovincialis* exposed to different treatments stained with hematoxylin. (i) Gills: cilia lost (*), hemocytes infiltration (arrows), evident enlargement of the central vessel (line with straight ends), abundance of lipofuscin aggregates (arrowheads); (ii) digestive glands: hemocytes infiltration (arrows), atrophied (a) and necrosis (n). Scale bar = 50 µm.

Figure 6 shows the gill’s histopathological alterations among treatments. The hemocyte infiltration (arrows) was found in all mussels’ tissues, especially in Hg and RSW conditions. Furthermore, evident enlargement of the central vessel (long arrows) was observed in organisms exposed to GO–PEI and RSW. Abundance of lipofuscin aggregates (*) in gills for each condition was observed. In digestive tubules, significantly higher *ih* values were observed in organisms exposed to Hg in comparison with the remaining treatments (Figure 5B). No significant differences were found among mussels exposed to CTL 21, GO–PEI, GO–PEI + Hg and RSW (Figure 5B, Table 3). Figure 6 also shows the digestive gland’s histopathological alterations among the conditions. The hemocyte infiltration (arrows) was found in all conditions, especially in mussel tissue exposed to Hg. Furthermore, atrophy (a) in digestive glands was shown in RSW and Hg conditions, in addition to necrosis (n).

### 3.4. Integrated Biomarker Response

The highest IBR value (2.09) was found on the mussels exposed to GO–PEI, while the lowest value (0.08) was observed in CTL 21 organisms. Moreover, the results obtained for organisms exposed to Hg, GO–PEI + Hg and RSW were 0.92, 0.57, 0.42 (respectively).

### 3.5. Multivariate Analysis

**Figure 7:**
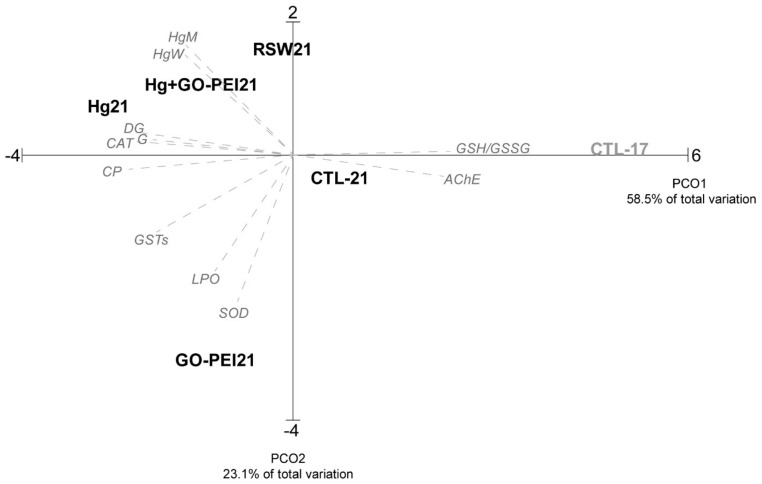
Principal coordinated analyses (PCO) based on Hg concentration, biochemical responses and histological alterations measured in *Mytilus galloprovincialis* exposed to different treatments (CTL 17, exposure at 17 °C; CTL 21, GO–PEI, GO–PEI + Hg, Hg and RSW, exposure at 21 °C) at the end of the experiment (28 days). Pearson correlation vectors are superimposed as supplementary variables (r > 0.75): ETS, SOD, CAT, GSTs, LPO, PC, GSH/GSSG, AChE, HgW, HgM, G (*ih*) and DT (*ih*).

The PCO graph based on Hg bioaccumulation in water and mussels and the biochemical and histopathological alterations is presented in Figure 7, revealing that PCO axis 1 explained 58.5% and PCO axis 2 explained 23.1% of the total variation. PCO1 separated the positive side mussels under control conditions (clean seawater at 17 °C) from the remaining conditions on the negative side. PCO2 clearly separated organisms exposed to GO–PEI on the negative side from the Hg, GO–PEI + Hg and RSW on the positive side. Mussels exposed to CTL 17 present a high correlation with GSH/GSSG and AChE (*p* > 0.9), while mussels exposed to Hg showed a high correlation with histopathological indices and CAT activity (*p* > 0.85). Mussels exposed to GO–PEI presented a high correlation with SOD (*p* > 0.88).

## 4. Discussion

In aquatic systems, climate warming and the presence of pollutants has been recently a topic of concern due to the scarce information on the combined impacts induced to inhabiting wildlife. Although efforts have been made to reduce the impacts of pollutants, namely through the development of water remediation processes, little is known on the effects of decontaminated water, especially if considering predicted warming scenarios. To increase the knowledge on this subject, in the present study, the impacts of temperature were addressed in non-contaminated and contaminated mussels exposed to contaminated and remediated seawater. The results obtained were discussed comparing biological responses observed in *Mytilus galloprovincialis* exposed to the same treatments but at different temperatures: 17 °C [1] vs. 21 °C (present study). For this, alterations on mussels’ metabolic capacity, oxidative and neurotoxic status as well as histopathological alterations were compared in non-contaminated mussels maintained at 17 and 21 °C. Furthermore, the influence of temperature was discussed by comparing the toxicological effects observed at 21 °C in contaminated mussels and mussels exposed to remediated seawater (present study) with the effects induced in mussels maintained at 17 °C [1] under the same treatments. 

### 4.1. Impacts of Temperature in Mussels Exposed to Control Treatment

Recent studies highlighted the impacts of increased temperature in marine species, namely bivalves [27,62,66,90,91,92,93,94]. The results presented here are in agreement with previous findings, showing that in non-contaminated organisms, increased temperature (21 °C) led to oxidative stress and generated neurotoxicity in comparison to mussels at the control temperature (17 °C). In particular, activation of antioxidant and biotransformation defenses was observed in organisms maintained at 21 °C compared with the ones at 17 °C. Although enhancing their defense mechanisms under increased temperature, mussels showed protein damage (represented by higher protein carbonylation) and loss of redox homeostasis (showed by the decrease in the ratio GSH/GSSG) in comparison to organisms at 17 °C. In addition to oxidative stress, neurotoxicity was also observed in non-contaminated mussels maintained at increased temperatures. Furthermore, histopathological damages in gills and digestive tubules with the accumulation of lipofuscin and hemocytes infiltration were observed in non-contaminated mussels under 21 °C, indicating that the increase of temperature could be responsible for histological alterations. Previous studies also demonstrated that bivalves exposed to warming scenarios enhanced the activity of antioxidant and biotransformation enzymes, although cellular damage still occurred [95,96,97,98]. Regarding histopathological effects, studies conducted by Pandey et al. [99] also showed that temperature increase was responsible for the occurrence of impacts in bivalves, with cilia and hemocytes damage in mussels’ gills (*Lamellidens marginalis*).

### 4.2. Impacts of Temperature in Mussels Exposed to Hg Treatments 

Several studies have been focused on the toxic impact of classical pollutants, namely metals (e.g., Hg), in bivalves [28,91,100,101,102]. The present study demonstrated that, under warming conditions, mussels exposed to Hg accumulated similar concentrations of this metal both when exposed to Hg alone or combined with GO–PEI. These findings indicate that under 21 °C, the presence of GO–PEI did not avoid the accumulation of the metal. However, previous studies developed by Coppola et al. [1] demonstrated that at 17 °C, the concentration of Hg in mussels exposed to GO–PEI + Hg (27 ± 5 µg/g) was significantly lower than the concentration of this metal in mussels exposed to Hg alone (42 ± 11 µg/g), indicating that under the control temperature, the nanomaterials may prevent Hg accumulation. However, the present findings further demonstrated that values of Hg in mussels’ tissues were lower at 21 °C (both at GO–PEI + Hg and Hg conditions) in comparison to values found in mussels at the same treatments but at 17 °C [1]. Similarly, Coppola et al. [91] as well as Coppola et al. [26] demonstrated that *M. galloprovincialis* exposed to Hg alone under two different temperatures (17 and 21 °C) accumulated lower metal concentration under warming conditions (8.4 ± 1 µg/g at 17 °C vs. 1.7 ± 0.19 µg/g at 21 °C [91]; 12.9 ± 5.2 µg/g 17 °C vs. 8.5 ± 1.4 µg/g at 21 °C). Such findings can indicate that increased temperature may change an organism’s behavior, enhancing its capacity to avoid Hg entrance through filtration reduction. This may not be the case since both at 21 °C (present study) and 17 °C [1], the ETS activity was similar in mussels exposed to GO–PEI + Hg and Hg and, thus, mussels might have the same filtration capacity at both temperatures. In fact, the present study demonstrated that in the presence of Hg (both alone or in combination with GO‒PEI), mussels were able to maintain their metabolic activity compared with non-contaminated mussels at the same temperature (21 °C) but also compared with organisms maintained at 17 °C. Although previous studies conducted by Freitas et al. [27] demonstrated that warmer conditions might decrease ETS activity in Hg contaminated mussels, this study differed from the present one. While in Freitas et al. [27], mussels were maintained at warming conditions for 14 days after which they were contaminated with Hg at the same temperature, in the present study, mussels were maintained at warming conditions in the presence of Hg during the entire experimental period (28 days). Therefore, in the previous study [27], the exposure to Hg was in fact shorter, and animals were already “acclimated” to the increased temperature, which may have allowed the organisms to decrease their metabolism to avoid Hg accumulation. In the present study, Hg and increased temperature were tested together for 28 days, which prevented mussels from having the same response. This hypothesis is supported by the results obtained at 17 °C [1] that reported lower ETS activity in Hg contaminated individuals, indicating that under control temperature, mussels may have the capacity to reduce their metabolism to limit Hg accumulation and injuries, which is a strategy no longer valid when the stress is higher, i.e., increased temperature combined with the presence of Hg. Thus, lower Hg concentration at 21 °C may result from alterations in the contaminant behavior rather on the organisms’ response or from other defense mechanisms not evaluated in the present study. This should be further explored. In the present study, the lack of metabolic activation may explain the fact that mussels did not enhance their antioxidant defense mechanisms in the presence of Hg, presenting similar SOD and CAT activity compared with control mussels at the same temperature (21 °C). On the other hand, the previous study conducted by Coppola et al. [1] demonstrated that although mussels tended to show lower ETS activity in the presence of Hg comparing to CTL, contaminated mussels were able to activate their antioxidant defenses, regardless of the tested treatment (Hg, GO–PEI + Hg). Nevertheless, for the same treatments (GO–PEI + Hg, Hg), antioxidant and biotransformation enzymes activities were similar at 21 and 17 °C. Furthermore, Morosetti et al. [103] showed similar CAT and GSTs activities in *M. galloprovincialis* exposed to Hg contamination at the control (17 °C) and increased (22 °C) temperatures. The limited antioxidant capacity observed in bivalves exposed to Hg (Hg and GO–PEI + Hg conditions) was associated with greater cellular damage (high LPO and PC levels) and low GSH/GSSG values (in the case of Hg treatment). In fact, although contaminated mussels tried to eliminate Hg through the activation of GSTs (higher GSTs values were observed in Hg and GO–PEI conditions compared with CTL 21), this detoxification mechanism was not sufficient to avoid cellular damage and the loss of redox balance. Furthermore, in the present study, the exposure to Hg did not enhance the neurotoxic effects, with similar AChE activity in Hg and GO–PEI + Hg-exposed mussels compared with the control ones at the same temperature (21 °C) and with Hg-contaminated mussels maintained at 17 °C [1]. The oxidative stress observed in contaminated mussels was accompanied by histopathological alterations in gills and digestive tubules, with higher impacts in mussels exposed to Hg alone. These results may indicate that the presence of GO–PEI may have prevented histopathological alterations. Comparing the present findings with results obtained under the same treatments but at 17 °C [1], it is possible to observe that similar responses were observed regardless of the temperature, indicating a higher impact of Hg than temperature.

### 4.3. Impacts of Temperature in Mussels Exposed to GO–PEI Treatment

Recent research has been testing the use of nanomaterials for water remediation, including GO–PEI, with few studies showing the impacts of these materials on marine wildlife [1,33,34,35,102,103]. Previous studies [1,33] have already demonstrated that at the control temperature (17 °C), GO–PEI (10 mg/L) induced biochemical impacts (metabolism and oxidative status alterations) in mussels and clams, with greater impacts in mussels. The present study further revealed impacts on mussels caused by GO–PEI at 21 °C, leading to oxidative stress and neurotoxic impacts. In this case, although detoxification (GSTs) and antioxidant (SOD) enzymes increased their activities, LPO was still observed in mussels exposed to GO–PEI at 21 °C, accompanied by a lower GSH/GSSG ratio. As a result of higher LPO values, SOD and GSTs activities in mussels exposed to GO–PEI, the highest IBR was obtained in this condition. Comparing the present results with previous ones [1], we may hypothesis that warming conditions increased the impacts of GO–PEI when acting alone, which may result from the toxicity of the material and/or higher sensitivity of mussels to it. In fact, mussels exposed to GO–PEI under 21 °C showed higher oxidative levels, with higher enzymes activity (SOD, CAT and GSTs activities of 0.77 ± 0.12, 27.32 ± 1.93 and 0.13 ± 0.01 U/g FW, respectively) when compared with mussels exposed to GO–PEI but at 17 °C (SOD, CAT and GSTs activities of 0.62 ± 0.11, 10.70 ± 3.13 and 0.03 ± 0.003 U/g FW, respectively [1]), with greater cellular damage at 21 °C (117.6 ± 8.46 nmol MDA/g FW) than at 17 °C (31.42 ± 5.16 nmol MDA/g FW). Furthermore, De Marchi et al. [104] showed increased detoxification capacity in bivalves (*Ruditapes decussatus*) exposed to carbon nanotubes and increased temperature, although in this case, no LPO was observed, which may be due to a shorter exposure period (96 h). Furthermore, Coppola et al. [33] demonstrated that for *R. philippinarum,* the antioxidant capacity increased in the presence of GO–PEI at warming conditions (22 °C), while no LPO was observed compared to CTL (without GO–PEI). This may indicate that clams might prevent LPO more efficiently than mussels when under the combined effect of temperature and GO–PEI. In the presence of the GO–PEI, no neurotoxicity was observed, with similar AChE activity in mussels exposed to GO–PEI and to CTL 21. Especially in gills, histopathological alterations were observed in GO–PEI exposed mussels. 

### 4.4. Impacts of Temperature in Mussels Exposed to Remediated Seawater

Mussels exposed to remediated seawater (RSW) showed the highest ETS activity compared with the other treatments. Since electron transport systems are responsible for the generation of ROS, this could explain higher LPO levels observed in this treatment in comparison to non-contaminated mussels at 21 °C (CTL 21). Nevertheless, mussels exposed to RSW and CTL 21 treatments presented similar CAT, GSTs, PC, GSH/GSSG and AChE levels, indicating that organisms exposed to remediated seawater were experiencing similar stress levels to organisms exposed to non-contaminated water (CTL) at the same temperature (21 °C). Previous studies conducted by Coppola et al. [1] also demonstrated that under 17 °C Hg, remediated seawater induced similar impacts to mussels as clean seawater (CTL) at 17 °C. However, when comparing the impacts at 21 °C (present study) and 17 °C [1], organisms exposed to remediated seawater at increased temperature presented higher impacts than organisms at 17 °C. In particular, higher metabolic capacity, antioxidant and biotransformation activity was observed in mussels exposed to RSW at 21 °C compared with mussels exposed RSW but at 17 °C. These results indicate that predicted warming conditions may enhance the impacts caused by remediated seawater, which can result from changing organisms’ sensitivity to pollutants or increasing pollutants toxicity, even at vestigial levels. Considering that previous studies conducted with clams under the same treatments [33] showed no differences at two different temperatures (17 and 21°C), the present findings may indicate a species-specific response and, therefore, a higher influence of temperature on organism sensitivity than on pollutant toxicity. Nevertheless, the results obtained further demonstrated that, even at increased temperature, organisms under RSW presented fewer alterations than mussels exposed to contaminated conditions (Hg, GO–PEI + Hg), highlighting the positive effects of water remediation processes.

## 5. Conclusions

Overall, comparing temperatures, the results clearly demonstrate that mussels exposed to clean seawater (control treatment) at 21 °C were under higher stress conditions (i.e., greater oxidative stress and neurotoxicity) than mussels at 17 °C. At 21 °C, mussels exposed to remediated seawater (seawater previously contaminated with Hg and decontaminated during 24 h with GO–PEI, RSW treatment) presented fewer biochemical alterations than mussels exposed to Hg (Hg and GO–PEI + Hg treatments). Furthermore, at the same temperature (21 °C), mussels exposed to RSW and control treatments showed similar biochemical and histopathological alterations. Nevertheless, mussels exposed to RSW at 21 °C showed greater metabolic capacity and antioxidant and biotransformation enzymes activity than mussels under the same treatment but maintained at 17 °C, revealing the effect of temperature on the toxicity of RSW.

## Figures and Tables

**Table 1:** Experimental treatments: CTL: control; GO–PEI: graphene oxide (GO) functionalized with polyethyleneimine; Hg: mercury; RSW: remediated seawater.

Conditions	Description
CTL 17	Hg 0.0 µg/L + GO–PEI 0.0 mg/L at 17 °C
CTL 21	Hg 0.0 µg/L + GO–PEI 0.0 mg/L at 21 °C
GO–PEI	GO–PEI 10 mg/L + Hg 0.0 µg/L at 21 °C
GO–PEI + Hg	GO–PEI 10 mg/L + Hg 50 µg/L at 21 °C
Hg	Hg 50 µg/L + GO–PEI 0.0 mg/L at 21 °C
RSW	Remediated Seawater previously contaminated with Hg (50 µg/L) and decontaminated by GO‒PEI (10 mg/L) for 24 h at 21 °C

**Table 2:** Mercury concentration in: (i) seawater samples (µg/L) collected immediately after the weekly water renewal for each treatment (results correspond to the mean ± standard deviation of four weeks; 3 samples per treatment and per week); (ii) mussels’ soft tissues (µg/g) collected 28 days after the beginning of the experiment (results correspond to the mean ± standard deviation; 3 mussels per aquarium, 9 mussels per treatment). Different uppercase letters represent differences among the treatments. LOQ (limit of quantification) for PSA 10.025 Millennium Merlin was ≤ 0.01 µg/L.

Conditions	Hg Water Concentration	Mussel’s Hg Concentration
**CTL 17**	<LOQ	0.17 ± 0.027 ^A^
**CTL 21**	<LOQ	0.08 ± 0.03 ^B^
**GO–PEI**	<LOQ	0.09 ± 0.01 ^B^
**GO–PEI + Hg**	52.1 ± 2.2 ^A^	13.09 ± 4.502 ^C^
**Hg**	52.4 ± 2.8 ^A^	16.19 ± 1.052 ^C^
**RSW**	9.6 ± 1.5 ^B^	6.09 ± 1.73 ^D^

**Table 3:** *P*-values, F-value, and F (DFn, DFd) (degrees of freedom numerator and denominator) obtained by pairwise comparisons between treatments (CTL17, CTL21, GO–PEI, GO–PEI + Hg, Hg and RSW) for each biomarker: Electron transport system activity (ETS); superoxide dismutase activity (SOD); catalase activity (CAT); glutathione-S-transferases activity (GSTs); lipid peroxidation levels (LPO); protein carbonyl levels (PC); ratio between reduced and oxidized glutathione (GSH/GSSG); acetylcholinesterase activity (AChE) and histopathological index: gills; digestive tubules; significant differences (*p* < 0.05) are highlighted in bold.

	ETS	SOD	CAT	GSTs	LPO	PC	GSH/GSSG	AChE	Gills	Digestive Tubules
**CTL 17 vs. CTL 21**	0.1948	0.3809	0.3809	**0.0003**	0.0889	**0.0001**	**0.0001**	**0.0001**	**0.0321**	**0.0001**
**CTL 17 vs. GO‒PEI**	0.0654	**0.0093**	**0.0093**	**0.0001**	**0.0026**	**0.0001**	**0.0001**	**0.0014**	**0.0001**	**0.0001**
**CTL 17 vs. GO–PEI + Hg**	0.1849	0.6506	0.6506	**0.0001**	**0.0482**	**0.0001**	**0.0001**	**0.0001**	**0.0001**	**0.0001**
**CTL 17 vs. Hg**	0.7773	**0.0379**	0.0379	**0.0032**	**0.0001**	**0.0001**	**0.0001**	**0.0001**	**0.0001**	**0.0001**
**CTL 17 vs. RSW**	**0.0005**	0.2197	0.2197	**0.0002**	**0.0171**	**0.0001**	**0.0001**	**0.0001**	**0.0001**	**0.0001**
**CTL 21 vs. GO–PEI**	0.639	**0.0363**	0.0363	**0.0002**	**0.0024**	**0.0001**	0.4829	0.4065	**0.0001**	0.9999
**CTL 21 vs. GO–PEI + Hg**	0.9579	0.1049	0.1049	**0.0063**	**0.0272**	**0.0002**	0.8785	0.5988	**0.0001**	0.9999
**CTL 21 vs. Hg**	0.402	0.2774	0.2774	0.1064	**0.0001**	**0.0001**	**0.0042**	0.5498	**0.0001**	**0.0001**
**CTL 21 vs. RSW**	**0.0001**	**0.022**	0.022	0.1012	**0.0011**	0.8038	0.7682	0.1474	**0.0001**	0.9999
**GO–PEI vs. GO–PEI + Hg**	0.5827	**0.0023**	0.0023	**0.0065**	**0.0081**	0.9759	0.4029	0.1906	0.999	0.906
**GO–PEI vs. Hg**	0.2006	**0.0492**	0.0692	**0.0181**	**0.0186**	**0.0324**	0.1087	0.1784	**0.0001**	**0.0001**
**GO–PEI vs. RSW**	**0.0001**	**0.0014**	0.0014	**0.0001**	**0.0116**	**0.0011**	0.8561	0.951	0.2278	0.9101
**GO–PEI + Hg vs. Hg**	0.4002	**0.0001**	0.0001	0.8328	0.123	**0.0436**	**0.0045**	0.8413	**0.0001**	**0.0001**
**GO–PEI + Hg vs. RSW**	**0.0004**	**0.0478**	**0.0478**	0.1776	0.7441	**0.0023**	0.7046	**0.0001**	0.227	0.9116
**Hg vs. RSW**	**0.0004**	**0.0001**	0.0001	0.4525	0.094	**0.0001**	0.2458	**0.0009**	**0.0001**	**0.0001**
*F–value*	*6.8*	*7.7088*	*22.168*	*13.823*	*10.034*	*71.835*	*56.966*	*13.846*	*47.304*	*39.377*
*F (DFn, DFd)*	*5, 48*								*5, 12*	

## Data Availability

All data generated or analysed during this study are included in this published article [and its Supplementary Materials Files].

## Supplementary Materials

The following are available online at https://www.mdpi.com/article/10.3390/nano11081978/s1.
