# Supplementary material for: The Influence of Temperature Increase on the Toxicity of Mercury Remediated Seawater Using the Nanomaterial Graphene Oxide on the Mussel Mytilus galloprovincialis
Source: Nanomaterials (Basel). 2021 Jul 31;11(8):1978. doi: 10.3390/nano11081978 (PMC8400667; doi:10.3390/nano11081978)
Supplement: Supplementary file 1 [file nanomaterials-11-01978-s001.zip › nanomaterials-1241730-supplementary.pdf]

# SUPPLEMENTARY MATERIAL

*Metabolic capacity*

The electron transport system (ETS) activity was measured based on King and Packard (1975) and the modifications performed by De Coen and Janssen (1997). Absorbance was measured during 10 min at 490 nm with intervals of 25 s and the extinction coefficient (Ɛ) of 15.9 $(mmol/L)^{-1}$ $cm^{-1}$ was used to calculate the amount of formazan formed. Results were expressed in nmol per min per g FW.

*Antioxidant enzymes activity*

The activity of superoxide dismutase (SOD) was quantified by following the method of Beauchamp and Fridovich (1971). The standard curve was obtained using SOD standards (0.25 – 60 U/mL). Absorbance was measured at 560 nm after 20 min of incubation at room temperature. The activity was expressed in U per g FW, where one unit (U) represents the quantity of the enzyme that catalyses the conversion of 1 µmol of substrate per min.

The activity of catalase (CAT) was quantified according to Johansson and Borg (1988). The standard curve was obtained using formaldehyde standards (0–150 µmol/L). Absorbance was measured at 540 nm. The enzymatic activity was expressed in U per g of FW, where U represents the amount of enzyme that caused the formation of 1.0 nmol formaldehyde per min at 25 °C.

*Biotransformation isoenzymes activity*

The activity of glutathione S-transferases (GSTs) was quantified following Habig et al. (1974). Absorbance was measured at 340 nm and the enzymatic activity was determined using Ɛ = 9.6 $(mmol/L)^{-1}$ $cm^{-1}$. The activity was expressed in U per g FW, where U is defined as the amount of enzyme that catalysis the formation of 1 µmol of dinitrophenyl thioether per min.

*Extent of cellular damage*

Levels of lipid peroxidation (LPO) were determined following the method described by Ohkawa et al. (1979). LPO levels were measured trough the quantification of malondialdehyde (MDA), a by-product of lipid peroxidation. Absorbance was measured at 535 nm and the extinction coefficient Ɛ = 156 $(mmol/L)^{-1}$ $cm^{-1}$ was used to calculate LPO levels, expressed in nmol of MDA formed per g of FW.

Protein carbonyl (PC) levels were obtained following Mesquita et al. (2014) protocol. Absorbance was measured at 450 nm and the extinction coefficient Ɛ = 22.308 $(mmol/L)^{-1}$ $cm^{-1}$ was used to calculated PC levels, expressed in nmol per g of FW.

*Redox balance*

Reduced glutathione (GSH) and oxidized glutathione (GSSG) were used as standards (0–60 µmol/L) and absorbance was measured at 412 nm (Rahman et al., 2007). The calculation of the ratio between oxidized and reduced glutathione contents was done considering the number of thiol equivalents (GSH/2 ∗ GSSG).

*Neurotoxicity*

Acetylthiocholine iodide (ATChI 5 mmol/L) substrates were used for the determination of acetylcholinesterase (AChE) activity following the method of Ellman et al. (1961). Enzyme activities were recorded continuously for 5 min at 412 nm. The activity was calculated using the extinction coefficient Ɛ = 13.6 $(mmol/L)^{-1}$ $cm^{-1}$ and expressed in nmol per min per g FW.
